# Supplementary figures and images for: A cluster-randomised controlled trial of the LifeLab education intervention to improve health literacy in adolescents
Source: PLoS One. 2021 May 5;16(5):e0250545. doi: 10.1371/journal.pone.0250545 (PMC8099135; doi:10.1371/journal.pone.0250545)

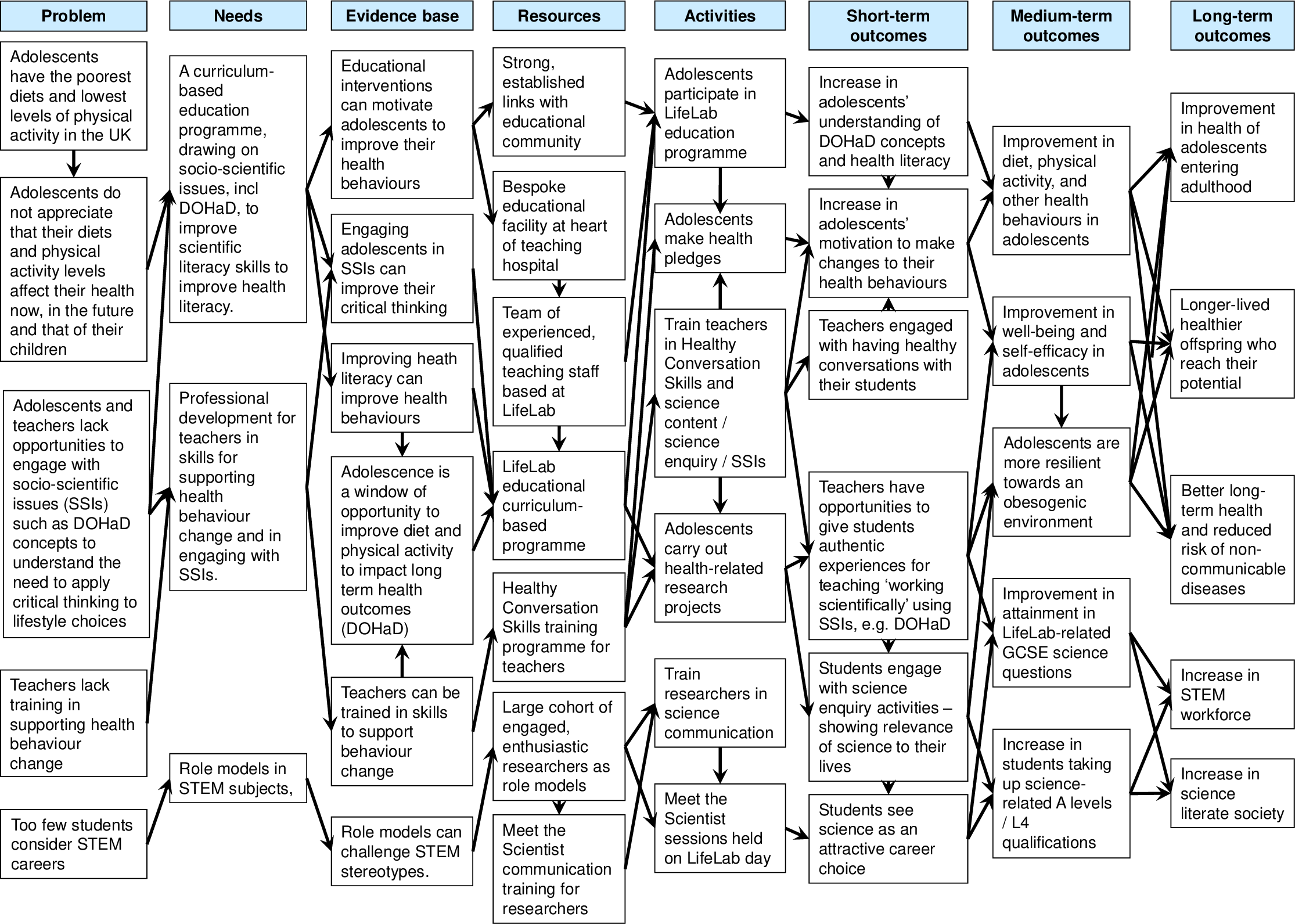

Supplement: S1 Diagram — (TIF) [file pone.0250545.s003.tif]
