# Supplementary material for: A cluster-randomised controlled trial of the LifeLab education intervention to improve health literacy in adolescents
Source: PLoS One. 2021 May 5;16(5):e0250545. doi: 10.1371/journal.pone.0250545 (PMC8099135; doi:10.1371/journal.pone.0250545)
Supplement: S1 File — (DOCX) [file pone.0250545.s004.docx]

**November 2014**

**SSEGM ETHICS SUB-COMMITTEE APPLICATION FORM**

***Please note:***

- ***You must not begin your study until ethical approval has been obtained.***
- ***You must complete a risk assessment form prior to commencing your study.***
- ***It is your responsibility to follow the University of Southampton’s Ethics Policy and any relevant academic or professional guidelines in the conduct of your study. This includes providing appropriate information sheets and consent forms, and ensuring confidentiality in the storage and use of data.***
- ***It is also your responsibility to provide full and accurate information in completing this form.***

**1. Name(s):** Prof Marcus Grace, Dr Janice Griffiths, Dr Kath Woods-Townsend, Dr Andri Christodoulou

**2. Current Position** Marcus Grace is Professor of Science Education

**3. Contact Details:**

**Division/School** Southampton Education School

**Email** mmg1@soton.ac.uk

**Phone** X23213

**4. Is your study being conducted as part of an education qualification?**

**No**

**5. If Yes, please give the name of your supervisor**

**6. Title of your project:**

Assessment of LifeLab Southampton: engaging teenagers in increasing their interest in science to improve their health behaviours (**BHF Project Grant no. PG/14/33/30827)**

**7. What are the proposed start and end dates of your study?**

**10 November 2014 – 30 September 2017**

**8. Describe the rationale, study aims and the relevant research questions of your study**

This project is essentially a continuation of the *LifeLab* project funded by Bupa “Achieving sustainable health behaviour change in adolescents”, and supported by the University’s RGO (RGO Ref: 7892). This new application is submitted with new funding from the British Heart Foundation to expand the number of pupils being surveyed in the randomised control trial evaluation of the project. The range of activities remains the same.

Over the last five years, the *LifeLab* project at Southampton has established an educational programme for 12-15 year olds, focusing on developing their understanding of the science underpinning non-communicable disease (NCD) risk in themselves and their future children. Our pilot studies have shown changes in their attitudes and health-related behaviours 6-12 months later.

The project objective is a formal evaluation of the efficacy of this existing intervention, in this age group, in producing attitude and behaviour change sustained for up to 12 months, and gaining further evidence on whether this intervention in adolescents is also associated with improved health behaviours and attitudes in their families.

Our primary objective is to measure effects of the intervention 12 months later, in students

aged 12-15 years, in terms of change in knowledge of NCD risk factors, including the impact of early

life environment, and attitude and behaviour in relation to diet and lifestyle, and their effects on the

students' health and that of their future children.

Secondary objectives are to assess (1) adolescents' abilities as science and health communicators

and potential change agents within their families and (2) whether the intervention induces change in

knowledge and attitude towards diet, physical activity and lifestyle in their parents.

During the project we will obtain additional data on the influence of gender and age at which the

intervention is applied (from 12-15 years) on outcomes.

**9. Describe the design of your study**

Our LifeLab intervention with school students already exists and has been tried and tested by the same project team over five years. It consists of curriculum-linked modules designed for 12-15 year olds, integrated with school science programmes and focusing on NCD risk and relevant science and health concepts. Key to this approach are hands-on visits to dedicated laboratories in a university/ hospital research setting. We will compare control and intervention groups by matched pre- and post-intervention validated questionnaires, to measure change in knowledge, attitude and behaviour in adolescents and their families. We will recruit 32 schools in or near Southampton and then randomly allocate 16 schools to receive the intervention; the other schools will act as controls. The control schools will only take part in the completion of questionnaires. Within each intervention school, three Year 9 classes of about 30 students each will be chosen for the intervention. The classes selected will be middle or mixed ability in science. A professional development programme, which we currently use, will be applied to ensure that the science teachers involved in the trial have adequate opportunity to engage with the pedagogy and the underlying science and plan the use of the learning resources in a manner appropriate to the knowledge and skills of the target classes.

The intervention will consist of the following components, all of which have already been tried and tested by the project team over the past five years:

• 4-6 week modules of work for use in Year 9;

• teacher professional development workshops relating to science and science education relevant to

implementation of the modules and including access to online support materials which describe the

underpinning science;

• a single day (5 hour) LifeLab hands-on programme, conducted within the setting of a customised

university/ hospital research lab, held part way through the module of work. Students interact

with real stories of science and current data, meet in small groups with scientists, and experience

first-hand activities utilising resources not available in a school laboratory. Examples of these activities

include using data loggers to measure the effect of exercise on heart rate and

blood pressure, measurement of arterial blood-flow and carotid artery wall thickness, bone density,

muscle strength, placental transport and extraction of their own DNA to explore the effect of lifestyle

on gene expression. Students and teachers who attend the LifeLab hands-on sessions will also be asked to complete a short evaluation sheet at the end of the day (this is also entirely voluntary).**These are all wholly non-invasive activities.**

The *LifeLab* project management group will provide a steer for the work. A project manager with a science teaching background will be appointed to drive the programme, and a research assistant will be appointed to develop and analyse evaluative work. Formative and summative evaluation procedures will be agreed, and perceived opportunities and barriers discussed to decide the most effective detailed approach to achieving project objectives.

The project manager will develop the school-based and university laboratory-based activities. Science teachers will deliver and manage school-based activities. The school-based part of programme will consist of a combination of whole class, group and individual student-centred tasks.

The project manager will collate and interpret questionnaire information and write a report for the funders on the overall findings and recommendations in conjunction with project management group.

**10. Who are the research participants?**

- About 2880 12-15 year old pupils from local schools
- The pupils’ teachers (about 50)

**11. If you are going to analyse secondary data, from where are you obtaining it?**

Southampton Women’s Survey – the SWS project team work alongside the *LifeLab* team, and the project is recognised as mutually beneficial.

**12. If you are collecting primary data, how will you identify and approach the participants to recruit them to your study?**

*Please attach a copy of the information sheet if you are using one – or if you are not using one please explain why.*

Participant Information sheets are attached – for pupils, parents and teachers

**13. Will participants be taking part in your study without their knowledge and consent at the time (e.g. covert observation of people)? If yes, please explain why this is necessary.**

No

**14. If you answered ‘no’ to question 13, how will you obtain the consent of participants?**

*Please attach a copy of the consent form if you are using one – or if you are not using one please explain why.*

Participant consent forms attached

Following lengthy discussion with the schools in and around Southampton, and in particular the schools recruited to this project, an opt-out consent procedure is suggested for the control pupils. Schools find the burden of having to collect, collate, chase up the consent forms too heavy. On average, 1/3 of parents were having to be phoned by the teachers in order to chase these consent forms, which is too great a burden for the schools to take on board. Schools are happy to take on the responsibility of an opt-out consent procedure, and see the health questionnaires as an activity which they would be happy to carry out within the science lessons.

**15. Is there any reason to believe participants may not be able to give full informed consent? If yes, what steps do you propose to take to safeguard their interests?**

No. We have engaged schools in these activities for five years without any problems gaining full informed consent from participants, please see point 14 for a discussion about the consent protocol we propose to adopt for the control schools. It has never been problematic ensuring informed consent for the intervention schools, as the students are coming out of school, the problems arise with the control schools - please see our proposal above.

**16. If participants are under the responsibility or care of others (such as parents/carers, teachers or medical staff) what plans do you have to obtain permission to approach the participants to take part in the study?**

Participant consent forms go to pupils’ parents/carers via the schools. They are signed and returned to us via the schools.

**17. Describe what participation in your study will involve for study participants. Please attach copies of any questionnaires and/or interview schedules and/or observation topic list to be used**

Pupils will take part in the school-based and the one-day university/hospital-based activities. All pupils, will be asked to complete pre and post-intervention questionnaires (attached).

**18. How will you make it clear to participants that they may withdraw consent to participate at any point during the research without penalty?**

All participants will be reminded clearly on questionnaires that they are free to withdraw from any activity or completion of any questionnaires at any time. The pupils will also be told this by their teachers prior visiting the labs.

**19. Detail any possible distress, discomfort, inconvenience or other adverse effects the participants may experience, including after the study, and you will deal with this.**

The activities are carefully planned to cause minimal stress to any participant. However, as the theme is health-related it is always possible that pupils might relate the work to their personal lives and become stressed as a consequence. The teachers involved are experienced and sufficiently expert to recognize stress among individual pupils, they will frequently remind pupils that they can withdraw from activities at any time, and there are no judgments made at any time about individual pupils’ health-related issues. The work centres around measuring the health of an imaginary pupil, which helps ‘depersonalise’ the activities, and divert attention away from individual pupils in the class.

**20. How will you maintain participant anonymity and confidentiality in collecting, analysing and writing up your data?**

All participants’ data will be stored anonymously following University guidelines. Prior to any student visits to laboratories full risk assessments will be conducted. It will also be made clear on questionnaires that all information will remain anonymous and confidential at all times. However, on the consent forms we include the text: We will record identifying information on the questionnaires. This is only so that we can match pre and post questionnaires. Once the questionnaire responses have been matched any identifying information will be deleted and an ID number assigned.

**21. How will you store your data securely during and after the study?**

All questionnaire data will be kept in accordance with the Data Protection Act and University policy. It will be stored in password protected areas on computer by the research team and only accessible by them.

**22. Describe any plans you have for feeding back the findings of the study to participants.**

An anonymised summary report will be produced for interested participants.

**23. What are the main ethical issues raised by your research and how do you intend to manage these?**

The activities are carefully planned to cause minimal stress to any participant. However, as the theme is health-related it is always possible that pupils might relate the work to their personal lives and become stressed as a consequence. The teachers involved are experienced and sufficiently expert to recognize stress among individual pupils, they will frequently remind pupils that they can withdraw from activities at any time, and there are no judgments made at any time about individual pupils’ health-related issues. The work centres around measuring the health of an imaginary pupil, which helps ‘depersonalise’ the activities, and divert attention away from individual pupils in the class.

**24. Please outline any other information you feel may be relevant to this submission.**

n/a
