# Supplementary material for: A cluster-randomised controlled trial of the LifeLab education intervention to improve health literacy in adolescents
Source: PLoS One. 2021 May 5;16(5):e0250545. doi: 10.1371/journal.pone.0250545 (PMC8099135; doi:10.1371/journal.pone.0250545)
Supplement: S2 File — (PDF) [file pone.0250545.s005.pdf]

Woods-Townsend *et al. Trials* (2015) 16:372
DOI 10.1186/s13063-015-0890-z

TRIALS

STUDY PROTOCOL Open Access

Engaging teenagers in improving their health behaviours and increasing their interest in science (Evaluation of LifeLab Southampton): study protocol for a cluster randomized controlled trial

Kathryn Woods-Townsend[1,2], Lisa Bagust[1], Mary Barker[3], Andri Christodoulou[1], Hannah Davey[1], Keith Godfrey[2,3], Marcus Grace[1], Janice Griffiths[1,4], Mark Hanson[5] and Hazel Inskip[3*]

Abstract

Background: Lifestyle and health behaviours are strongly linked to non-communicable disease risk, but modifying them is challenging. There is an increasing recognition that adolescence is an important time for lifestyle and health behaviours to become embedded. Improving these behaviours in adolescents is important not only for their own health but also for that of their future children. LifeLab Southampton has been developed as a purpose-built classroom and laboratory in University Hospital Southampton. Secondary school students visit LifeLab to learn how childhood, adolescent and parental nutrition influences health, understand the impact of their lifestyle on their cardiovascular and metabolic health, and to inspire them with the excitement of research and future career possibilities in science. The LifeLab visit is part of a programme of work linked to the English National Curriculum. Pilot work has indicated that attitudes towards health can be changed by such LifeLab sessions.

Methods/Design: A cluster randomised controlled trial is being conducted to evaluate the effectiveness of the LifeLab intervention, the primary outcome being a measurement of the change in nutrition, health and lifestyle literacy from before to after the LifeLab intervention.

The LifeLab intervention comprises professional development for the teachers involved; preparatory lessons for the school students, delivered in school; a hands-on practical day at LifeLab, including a 'Meet the Scientist' session; post-visit lessons delivered in school; and the opportunity to participate in the annual LifeLab Schools' Conference. This study aims to recruit approximately 2,500 secondary school students aged 13 to 14 years from 32 schools (the clusters) from Southampton and neighbouring areas. Participating schools will be randomised to control or intervention groups. The intervention will be run over two academic school years, with baseline questionnaire data collected from students at participating schools at the start of the academic year and follow- up questionnaire data collected approximately 12 months later.

Trial registration: Evaluation of LifeLab is a cluster randomised controlled trial (ISRCTN71951436, registered 25 March 2015), funded by the British Heart Foundation (PG/14/33/30827).

Keywords: science education, health literacy, science literacy, nutrition literacy, cluster randomised trial, adolescent health, health behaviours, LifeLab

* Correspondence: hmi@mrc.soton.ac.uk
[3]MRC Lifecourse Epidemiology Unit, University of Southampton, Southampton, UK
Full list of author information is available at the end of the article

© 2015 Woods-Townsend et al. **Open Access** This article is distributed under the terms of the Creative Commons Attribution 4.0 International License (http://creativecommons.org/licenses/by/4.0/), which permits unrestricted use, distribution, and reproduction in any medium, provided you give appropriate credit to the original author(s) and the source, provide a link to the Creative Commons license, and indicate if changes were made. The Creative Commons Public Domain Dedication waiver (http://creativecommons.org/publicdomain/zero/1.0/) applies to the data made available in this article, unless otherwise stated.

Background

There is a growing agenda for the promotion of a healthy lifestyle in young people for the reduction in the risk of cardiovascular and other non-communicable diseases [1–4]. Non-communicable diseases (NCDs) place a heavy burden on society and on hospital and community-health services; their prevention not only benefits the individuals at risk, but reduces the pressure on limited health resources. Our research in Southampton has focused on the processes by which the developmental environment affects later risk of ill health such as obesity or NCDs. The insights from this research are highly relevant to today's society, because they raise issues about personal choice, responsibility for health and the need for better informed decisions about diet, lifestyle and the ethical dilemmas faced by technological societies. The Shanghai Declaration of the Worldwide Universities Network, led by the University of Southampton and prepared at the request of the World Health Organisation ahead of the 2011 United Nations high-level meeting of the General Assembly on the prevention and control of non-communicable diseases (NCDs), concluded that 'Particular attention should be paid to both population- and individual-based approaches to increase access to education, to promote health literacy in children, adolescents and parents, … to both reduce the burden of NCDs and provide other benefits' [2] and The Lancet Series on Adolescent Health [5] states that the 'Strongest determinants of adolescent health are national wealth, income inequality and access to education'.

There are great inequalities in dietary quality and the adoption of healthy lifestyles in the UK. Southampton is a relatively deprived city in the South of England, and it falls in the 25 % most deprived local authorities in England and Wales. The Southampton Women's Survey (SWS) is an ongoing, prospective cohort study which started in 1998 to assess preconception characteristics of a general population sample of young women and to investigate a wide range of maternal influences on pregnancy outcomes and child health. A detailed description of the rationale, study design and protocol has been published elsewhere [6]. Dietary data from the SWS [6] were analysed using principal components analysis. The first component of the analysis of the women's data can be interpreted as a 'prudent' diet score: this score summarises the degree to which each woman complies with a prudent/healthful dietary pattern, characterised by high intakes of fruit, vegetables, whole grain, fish and poultry [7]. This pattern has been found to be positively associated with micronutrient intake [7] and negatively associated with saturated fat intake [8]. A striking finding from the SWS is the strong and graded relationship between women's educational attainment and their prudent diet score, showing for example that among women who left school with no educational qualifications, 55 % had a prudent diet score in the lowest quarter of the distribution in contrast to only 3 % of those women with degree level qualifications [9]. Analysis of other lifestyle data from the SWS has also shown great differences across the population, with higher levels of obesity and smoking and lower levels of physical activity in the most disadvantaged areas of the City [10].

In Southampton, we have shown that women change their diets and lifestyles only marginally when planning a pregnancy and during pregnancy itself [11, 12], and thus lifestyles adopted earlier in life have long-term consequences both for women themselves and for their children. Importantly, dietary patterns established by women before they become pregnant [13, 14] are strongly associated with the way in which they feed their children in infancy and childhood, and in turn with the health and development of the child [15–17]. Women's diets, however, are also influenced by their partners, and fathers play an important role in determining the diet and lifestyle of the family [18], which reflects on their own cardiovascular health too.

The effects of a woman's diet and general health as she embarks on pregnancy can have profound and lasting effects on both early development and the lifelong health of her children [19–23]. Specifically, unhealthy lifestyles in mothers, including unbalanced diets, unhealthy body composition, excessive stress and low levels of physical activity, have been associated with increased risks in their children in later life of heart disease, diabetes, poor respiratory health and osteoporosis [24, 25]. A recent review of six European studies concluded that interventions are needed before, as well as during, pregnancy to improve the diets of families with young children [26].

Health literacy is considered as 'a means to enabling individuals to exert greater control over their health and the range of personal, social and environmental determinants of health' [27]. This view of health literacy takes into account the important role of educating individuals about their health and enabling them to develop the critical and evaluative thinking skills required to make informed health-related decisions, and develop healthy behaviours which are sustained across their lifetime [28]. The development of such critical thinking skills and the ability to consider and weigh evidence are part of core scientific practices and one of the advantages that science education has to offer to the general education of young individuals [29].

We aim to promote health literacy through science education. Science education is important not only because it provides the basis for the development of future scientists but also because it can sustain and improve the scientific literacy of the wider population [30].

Scientific literacy describes both the ability to understand scientific content and the ability to engage in critical evaluation and discussions about scientific issues as these present themselves in everyday life [31]. Knowledge of scientific principles, especially those related to human biology, critical thinking dispositions and the ability to make informed decisions about science related issues, are attributes closely linked to health literacy.

The evidence points to a need to improve health and nutrition literacy to promote healthy lifestyles in young people in order to prevent cardiovascular disease in them and in their children and to reduce health inequalities. Part of the solution to these problems lies in education. We have undertaken a systematic review of approaches to behaviour change interventions, and it is clear that successful interventions include educational components [32]; education can change attitudes, alter health-related behaviours and increase health literacy in young people [33, 34].

Interventions targeting teenagers have a double advantage. During adolescence health behaviours are being developed and established, and improving them at this age should lower the risks of later chronic disease. Additionally, however, such improvements in health behaviours would enable them to be better prepared when they embark on having their own children.

Interventions with groups of school classes have been shown to play a key role in helping to prevent the adoption of high-risk health-related behaviours [35]. These considerations have led us to collaborate with schools in Southampton and Hampshire to develop a classroom project, LifeLab, embedded within the new Southampton Centre for Biomedical Research (SCBR) at UHS. As many young people have never been inside a hospital or visited a research laboratory, such an experience can make a great impression. Indeed, learning outside the classroom has been recognised as often providing the most memorable learning, with experiences that are remembered into adulthood and affect behaviour, lifestyle and work [36–40].

The theme of LifeLab is ***Me, My Health & My Children's Health***. The teaching programme is tailored to reach students of all abilities and is primarily aimed at secondary school students aged 13–14 years. It includes professional development for all teachers involved in the project, a series of before and after lessons delivered in school and a hands-on practical activity day in the LifeLab facility. The school lessons are explicitly linked to the UK National Curriculum and embed the messages of the LifeLab visit. During the LifeLab activity day, students have opportunities to carry out hands on practical activities designed to build on the lessons they have been taught in school. The programme also has scope for training teenagers in cardio-pulmonary resuscitation (CPR) techniques, enabling them to play a part in reducing cardiovascular mortality and also providing strong messages about the consequences of failure to prevent cardiovascular disease. Pilot work showed that participation in a science programme focusing on health, and experiencing learning within a hospital-based classroom had a positive influence on teenagers' awareness of the importance of making healthy lifestyle choices [37]. Currently, a small scale evaluation of the LifeLab intervention is being undertaken in six schools. However, as LifeLab is an intervention to improve knowledge, attitudes and behaviour in young people from across society, a large-scale cluster randomised control trial is now being conducted. While our particular focus is on the prevention of cardiovascular disease and other NCDs this type of educational approach needs to be fully assessed to see if does have an impact on the health behaviours of young people.

Aim

The aim of this cluster randomised controlled trial is to evaluate whether an educational intervention in the form of LifeLab targeting teenagers improves the following in the teenagers:

1. nutrition, health and lifestyle literacy.
2. ability to use CPR techniques.
3. health behaviours with respect to diet and lifestyle.
4. understanding of the long-term influences of their health behaviours on their subsequent health and that of their future children.
5. self-efficacy in relation to diet and lifestyle.
6. engagement with science (intention to study science post GCSE/ pursue a career in a science-related job).

Methods/Design

Trial design

The target population for the evaluation is the state secondary schools in Southampton and surrounding areas. We propose to evaluate LifeLab through a cluster randomised control trial, recruiting Year 9 students (aged 13 to 14 years) from 32 state secondary schools/academies (approximately 2,500 students) to take part in the project. Each school will be randomly allocated to either 'control' or 'intervention' status. During year 1 (school year 2014–15) we aim to recruit 16 schools; eight will therefore be intervention schools and their Year 9 students will take part in the LifeLab programme, whereas the other eight schools will form the control group. During year 2 (school year 2015–2016), this process will be repeated. Schools that are randomised to the control arm of this intervention will be invited to attend LifeLab in the academic year *following* the year they contribute to the control group. Questionnaires will be administered to

control and intervention students after recruitment (baseline questionnaires) and again approximately 12 months after the baseline questionnaires were completed. There may be some small loss to follow-up due to children moving schools during the school year, but we do not anticipate this having major impact; in our current small-scale trial, at the 12-month follow-up, 2 % of the students were unavailable to participate due to moving schools. We emphasise in our discussions with schools during the recruitment phase the need to track the students over the length of the trial and ask that schools provide us with the new school details for those students who have left. We will then contact the new schools to request permission to work with those students who have moved. Good working relationships with the schools have been developed over many years and should assist in minimising any dropout at the school level. The Heads of Science of all the Southampton and many Hampshire secondary schools are enthused by LifeLab and are committed to it. They have been involved from a very early stage and have always been keen for their students to take part in our taster and pilot activities. We envisage good take-up within the schools and do not foresee recruitment difficulties. We appreciate that the control schools may wish to receive the intervention straight away, but all control schools are offered participation in the following year. The flow of the trial is shown in the CONSORT diagram in Fig. 1.

Recruitment

Schools will be recruited through presentations at the Secondary Heads of Science forum meetings, building on previous excellent working relationships. All secondary state schools/academies are eligible for participation. We exclude participation of independent, grammar and special schools, as the population of students would be more selective and could potentially bias the results of the trial. The main approach will be through recruitment letters and phone calls to both the Head of Science and Head Teacher at all schools throughout our target region. The recruitment pack includes a letter of agreement which the Head Teacher is required to sign, and upon receipt of this letter a further meeting will be arranged with the Head of Science and a member of the Senior Leadership Team. At this meeting, the requirements of signing up to the randomised controlled trial will be clarified and the expectations of the school as either an intervention or a control school will be fully explained. It will also be an opportunity for the school to ask questions around implementation of the intervention for forward planning on their behalf. Schools are asked to allocate a minimum of three classes to participate in the trial (approximately 90 pupils), and we ask that these are their 'middle ability' students. As the teaching programme is planned for this age range and is differentiated for ability, there are no exclusion criteria for pupils. For pupils who may require more input (for example, English as a second language), we provide support for schools in planning the provision. This is discussed at the teachers' professional development day. Schools will already have provisions in place for these students, and so it is a matter of ensuring that the LifeLab materials are accessible by all students who will be likely to participate in each school. Following this meeting, if the school is still happy to participate it will be put through to the randomisation phase.

Randomisation procedure

Groups of recruited schools will be randomised in blocks of even numbers. Recruiting schools is an ongoing process and the smallest block size will be two, such that once a pair of schools is recruited, and if others are unlikely to be recruited soon, the pair will be randomised. Where possible, the block size will be larger. Once at least two schools have been recruited and the Head Teachers' letters returned, the schools are numbered and documented and dated. The randomisation is then conducted off-site by a statistician at the MRC Lifecourse Epidemiology Unit using computer-generated sequences, with no knowledge of the schools in question. Prior to randomisation, no matching of schools takes place. Schools require the information about their status in a timely fashion in order to plan their curriculum/allocate staff and resources in advance. Schools are recruited throughout the year, and it would be difficult to match schools by exact criteria. We are recruiting from a large area and a major consideration when calculating the numbers of schools necessary to recruit was to ensure a wide representation of schools/pupils.

Ethics approval and research governance

The study has been approved by the research ethics committee in the Southampton Education School, Faculty of Social and Human Sciences, University of Southampton (ERG reference: 12328). The study is funded by the British Heart Foundation (PG/14/33/30827) and has been registered on the ISRCTN database (ISRCTN71951436, registered 25 March 2015). The research sponsor is the University of Southampton. The LifeLab Directors provide oversight of the trial, acting as a trial steering committee, with reference to the LifeLab External Liaison Group for scrutiny.

Consent

Participant information sheets are sent with the initial recruitment pack to potential schools. Once the school has agreed to participate and signed the agreement letter and following the randomisation process, parent and

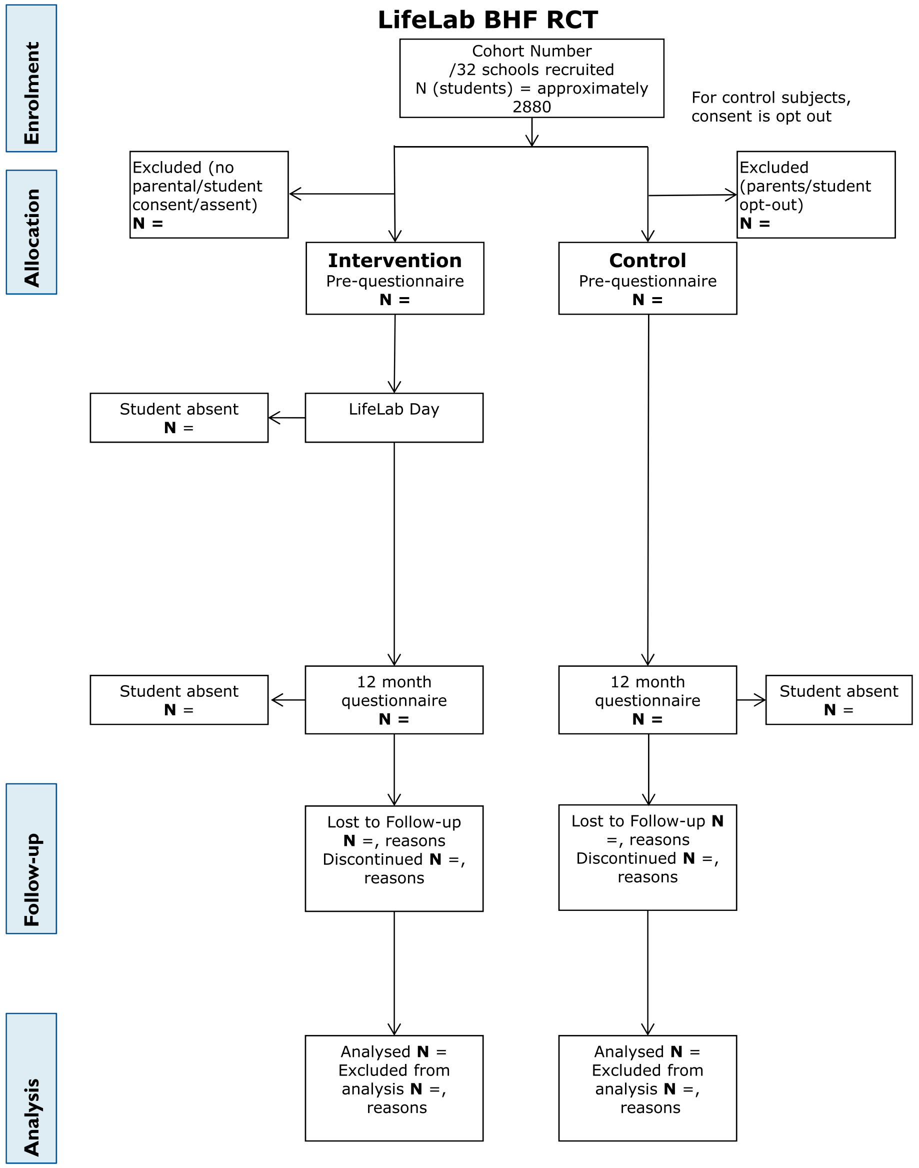

Fig. 1 Consort diagram to show the flow of participants through the trial. This CONSORT diagram shows the order and timing of the stages which the participants in the trial take part in

pupil information sheets are provided for the schools to disseminate to pupils and parents directly. Two versions of the information sheets have been produced - depending on whether the school is part of the 'intervention' or 'control' arm of the trial. Parents are provided with contact details of the research team in case they have questions about the LifeLab programme and associated research trial.

For the intervention schools, consent will be opt-in and collected prior to any research data being collected. For the control schools, consent will be opt-out. While not ideal, discussions with schools around this have been carried out, and the request from schools was that an 'opt-out' pathway was followed as this would place less of a burden on the control schools. Parent/pupil information sheets will be sent home, and parents who do not want their children to take part in the research questionnaires will be required to return the form indicating this.

In all cases, pupil assent is built into the questionnaire and, at any point, a pupil or parent can request that their information be withdrawn.

The intervention

The proposed intervention comprises the following:

1. Professional development for the teachers.
2. A 2 to 3 week module of work [41] for use with Year 9 school students (13 to 14 year olds), linked to the UK National Curriculum encompassing both before and after lessons to be delivered in school.
3. A hands-on practical day visit to LifeLab in the Southampton Centre for Biomedical Research at University Hospital Southampton, held part way through the module.

The materials for the work in school have been produced [41] and continue to be developed, and the activities for the LifeLab visit have been tested in our pilot work. Students will have opportunities to experience a variety of ways to measure health: assessing carotid artery blood flow and structure using ultrasound, measuring body composition, performing lung function tests, training in CPR and testing grip strength and flexibility. They are also able to extract their own DNA and carry out gel electrophoresis experiments that illustrate how a healthy diet can induce epigenetic changes that alter DNA structure and are passed from parents to offspring, with implications for cardiovascular and lifelong health for themselves and their children. Health messages are linked to the hands-on practical activities at LifeLab and to the school-based activities, to ensure that the teenagers understand the long-term implications of their current diet and lifestyle on their cardiovascular health.

Outcome measurements

Our primary outcome is a measure of nutrition, health and lifestyle literacy based on the critical nutrition literacy scale developed by Guttersrud et al. [42], adapted for use by teenagers and supplemented with broader lifestyle questions. Secondary outcomes will be the students' understanding of influences on their cardiovascular health and that of their subsequent children, their health behaviours such as dietary patterns [43], physical activity, their self-efficacy scores in relation to diet and lifestyle and their intent to continue studying science post-GCSE/seek a career in a science-related job. Summary statistics at the school level will be obtained. Information on the resources required to provide LifeLab will be obtained. Outcome measures are collected in schools using web-based questionnaires. The LifeLab team provides support to the teachers to administer the process, but the students complete the questionnaires independently on their computers. A written script is used in all schools to explain the process to the students to ensure consistency across all schools, both control and intervention (please see Additional file 1).

Data management

Questionnaire data will be collected via a website-based questionnaire (hosted by the University of Southampton). These data will be downloaded via a dedicated computer and stored on a University server. All data will be kept in accordance with the Data Protection Act, University of Southampton Data protection policy and in accordance with the protocols of the MRC Lifecourse Epidemiology Unit. It will be stored in password-protected areas on computer by the research team and only accessible by them. Data will be stored in Access databases and analyses conducted in Stata and SPSS. The data will be managed with support from the data management staff of the MRC Lifecourse Epidemiology Unit, which has extensive data management expertise and houses data from more than 200 studies.

Identifying information will be collected about participants, purely for the purposes of matching pre- and post-questionnaires. All identifying data will be removed from the rest of the data after linkage is complete and will be stored separately. It will only be kept in case there is future research in which a follow-up is planned.

Process evaluation

The intervention will be accompanied by a full and detailed process evaluation to include assessment of the context, fidelity, implementation and reach of the intervention, and barriers to implementation [44, 45]. It will include an assessment of contamination of control schools where science teachers have moved from intervention schools. Oversight of the process evaluation will be led by a health psychologist (MB) who has extensive experience in the field of process evaluation and was involved in the recent MRC publication of guidelines for process evaluation [46]. She is independent from the implementation of the intervention and so can provide an unbiased oversight. An education researcher (AC) will be responsible for conducting observations in schools to ensure fidelity to the teaching programme and whilst she is involved in the professional development for the school teachers, in order to establish a credible relationship with the education community, she is independent of the implementation of the intervention from that point onwards.

Statistical analyses

We will perform an intention-to-treat analysis using multi-level models for the quantitative data. Specifically, random effects models will be used to take account of the clustered nature of our data. Multi-level logistic (for rare outcomes) or Poisson regression models with robust variance (for more common outcomes) will be used for binary outcome variables and multi-level linear regression for continuous measures. The number of clusters is

relatively small, and summary analyses will be conducted at school level, not least as this will allow us to feedback school-level data to the schools that have been involved in the trial. Our endpoint will be the results of a 12-month follow-up with adjustment for baseline responses included in the modelling.

Planned subgroup analyses would focus on whether there are different effects for boys and girls. We will analyse the uptake of LifeLab in different ethnic groups and in those who are more disadvantaged. Compliance will also be assessed in relation to various confounding factors to assess the bias associated with the different consent procedures imposed on us by the schools for the intervention and control groups. Sensitivity analyses will be conducted as appropriate using sampling from the intervention arm to try to account for this bias. In practice, during our pilot work, as schools do not want children remaining in school when the rest of the class is out on a trip, very few children are not included in the intervention arm, and thus we are confident that there is likely to be little difference in response rates between the two arms of the trial.

Financial evaluation

These data will be combined with appropriate unit cost data to estimate the costs of LifeLab. We will use the health and nutrition literacy score as the outcome measure in a cost-effectiveness analysis and will estimate the cost per unit change in this score. These data will be important in determining the level of support needed to sustain LifeLab on a longer term basis.

Power calculations

Using data on behaviour (z-scores), validated in our previous research in young adults in and around Southampton [43], we have estimated an intra-class correlation of 0.035, but to be conservative we have rounded this up to 0.04. We propose to sample three Year 9 (aged 13 to 14 years) classes from each school, averaging 90 students from each school. Using 90 as our cluster size we estimate that with 14 clusters in each group, we will have 80 % power at the 5 % level of significance to detect a difference in change in our primary outcome score of 0.25 SDs from the beginning to end of the school year between the intervention and control schools. Comparable effect sizes have been considered in other health interventions as being meaningful in terms of the impact on health behaviours, and our level of 0.25 SDs falls in the mid-range of effect sizes reported in a meta-synthesis of meta-analyses of behaviour change interventions in the general population [47]. As described above, we will study 32 year groups, thus allowing some leeway for dropouts. To minimise loss of pupils to follow-up, we will request class lists for each participating class (both from the intervention and control schools), so that missing participants at each stage of the follow-up can be identified and asked to complete the questionnaires.

Discussion

A formal assessment of LifeLab would show the value of the programme in educating teenagers about their cardiovascular health and about the value of science. It would point to ways to improve and develop the LifeLab concept and identify the activities that have the most profound effect on the students. If shown to be successful, this would provide the impetus to sustain LifeLab in Southampton and encourage the development of similar initiatives in other cities in the UK and around the world. There are a few projects similar to LifeLab which have been established in other cities worldwide. The Liggins Institute was at the forefront of this work with LENScience [48], and Lord Winston established the Reach Out Lab at Imperial College, which, although a similar concept offering school students opportunities to experience science outside of the school environment, has different aims, focusing on motivating high attaining children to consider careers in science-related subjects. Plans are now being developed for laboratories elsewhere, similar to LifeLab, including in other countries such as Australia, South Africa and Ireland.

Improving the diets and lifestyles of young people is an important route both to reducing NCDs in their later lives but also improving the health of the next generation. By targeting children from all backgrounds this provides a form of societal intervention, such as has recently been advocated as likely to have a greater impact on cardiovascular disease inequalities than targeting those at high risk of the disease [49].

Trial status

Recruitment for the RCT has been initiated, and 18 schools have been recruited and have already progressed through the randomisation phase.

Additional file

Additional file 1: Pre-questionnaire script. (PDF 66 kb)

Abbreviations

NCD: non-communicable disease; RCT: randomised control trial; CPR: cardiopulmonary resuscitation; SWS: Southampton Women's Survey.

Competing interests

KMG has received reimbursement for speaking at conferences sponsored by nutrition companies and is part of an academic consortium that has received research funding from Abbott Nutrition, Nestec and Danone. The University of Southampton has received an unrestricted donation from Danone Nutricia to support LifeLab's work with schools.

Authors' contributions

KWT carried out the pilot work; was involved in the conception, design, development and delivery of the programme of work; and drafted the manuscript with help from HI and the other authors. LB and HD were involved in the design, development and delivery of the teaching programme of work, including the professional development programme for teachers. MEB was involved in the design of the cluster randomised trial and concurrent process evaluation. MEB, AC, MG and HI were involved in the design of the research questionnaires. AC, MG, JG, HI, KG and MH were involved in the initial design of the teaching programme and in the design and delivery of the professional development programme for participating teachers. MG, KG, JG, MH and HI have driven the development of LifeLab from its early origins in 2007, including finding funding and have designed the cluster randomised trial. All authors have contributed to the manuscript and approved the final submitted version.

Acknowledgements

We would like to thank the schools, teachers, students and parents who participate so enthusiastically in the LifeLab teaching programme. In conducting pilot activities for LifeLab we have collaborated closely with colleagues from the highly acclaimed Liggins Education Network for Science (LENS) project at the University of Auckland (www.auckland.ac.nz/uoa/liggins/lens/lens.cfm), and would like to extend thanks for their on-going support and continued useful discussions, in particular Jacquie Bay, director of LENScience. LifeLab, has been developed as a joint initiative, involving the University of Southampton and the University Hospital Southampton NHS Foundation Trust, the MRC Lifecourse Epidemiology Unit, the University of Southampton (UoS) Faculty of Medicine and Southampton Education School, the Mathematics and Science Learning Centre (MSLC), the NIHR Southampton Biomedical Research Centre in nutrition (BRC), and the secondary schools themselves, Southampton City Council and Hampshire County Council.
The cluster randomised trial is funded by the British Heart Foundation. LifeLab has also received research funding from the Wellcome Trust, Cancer Research UK, Research Councils UK, the BUPA Foundation, the Primary Science Teaching Trust (formerly the Astra Zeneca Science Teaching Trust), the EPSRC (via the UoS Pathways to Impact funding scheme). KWT & MAH are supported by the National Institute for Health Research through the NIHR Southampton Biomedical Research Centre. KMG is supported by the National Institute for Health Research through the NIHR Southampton Biomedical Research Centre and by the European Union's Seventh Framework Programme (FP7/2007-2013), project EarlyNutrition under grant agreement n° 289346. This report is independent research by the National Institute for Health Research Biomedical Research Centre Funding Scheme. The views expressed in this publication are those of the author(s) and not necessarily those of the NHS, the National Institute for Health Research or the Department of Health. The Garfield Weston Foundation gave a generous donation to the building of LifeLab.

Author details

[1]Southampton Education School, Faculty of Social and Human Sciences, University of Southampton, Southampton, UK. [2]NIHR Southampton Biomedical Research Centre in Nutrition, University Hospital Southampton, NHS Foundation Trust, Southampton, UK. [3]MRC Lifecourse Epidemiology Unit, University of Southampton, Southampton, UK. [4]Mathematics and Science Learning Centre, Southampton Education School, Faculty of Social and Human Sciences, University of Southampton, Southampton, UK. [5]Human Development and Health Academic Unit, Faculty of Medicine, University of Southampton, Southampton, UK.

Received: 1 April 2015 Accepted: 28 July 2015
Published online: 21 August 2015

References

1. Biro FM, Wien M. Childhood obesity and adult morbidities. Am J Clin Nutr. 2010;91:1499S–505S.
2. Hanson M, Gluckman P, Nutbeam D, Hearn J. Priority actions for the non-communicable disease crisis. Lancet. 2011;378:566–7.
3. Low FM, Gluckman PD, Hanson MA. Developmental plasticity and epigenetic mechanisms underpinning metabolic and cardiovascular diseases. Epigenomics. 2011;3:279–94.
4. Godfrey KM, Inskip HM, Hanson MA. The long-term effects of prenatal development on growth and metabolism. Semin Reprod Med. 2011;29:257–65.
5. Sawyer SM, Afifi RA, Bearinger LH, Blakemore SJ, Dick B, Ezeh AC, et al. Adolescence: a foundation for future health. Lancet. 2012;379:1630–40.
6. Inskip HM, Godfrey KM, Robinson SM, Law CM, Barker DJ, Cooper C. Cohort profile: the Southampton women's survey. Int J Epidemiol. 2006;35:42–8.
7. Kant AK. Dietary patterns and health outcomes. J Am Diet Assoc. 2004;104:615–35.
8. Crozier SR, Robinson SM, Borland SE, Inskip HM. Dietary patterns in the Southampton women's survey. Eur J Clin Nutr. 2006;60:1391–9.
9. Robinson SM, Crozier SR, Borland SE, Hammond J, Barker DJ, Inskip HM. Impact of educational attainment on the quality of young women's diets. Eur J Clin Nutr. 2004;58:1174–80.
10. Wilkinson R, Inskip H. The health and wellbeing of women in Southampton's communities: an analysis of the Southampton women's survey. 2006.
11. Crozier SR, Robinson SM, Godfrey KM, Cooper C, Inskip HM. Women's dietary patterns change little from before to during pregnancy. J Nutr. 2009;139:1956–63.
12. Inskip HM, Crozier SR, Godfrey KM, Borland SE, Cooper C, Robinson SM. Women's compliance with nutrition and lifestyle recommendations before pregnancy: general population cohort study. BMJ. 2009;338:b481.
13. Robinson SM, Marriott LD, Crozier SR, Harvey NC, Gale CR, Inskip HM, et al. Variations in infant feeding practice are associated with body composition in childhood: a prospective cohort study. J Clin Endocrinol Metab. 2009;94:2799–805.
14. Robinson S, Marriott L, Poole J, Crozier S, Borland S, Lawrence W, et al. Dietary patterns in infancy: the importance of maternal and family influences on feeding practice. Br J Nutr. 2007;98:1029–37.
15. Gale CR, Martyn CN, Marriott LD, Limond J, Crozier S, Inskip HM, et al. Dietary patterns in infancy and cognitive and neuropsychological function in childhood. J Child Psychol Psychiatry. 2009;50:816–23.
16. Brazionis L, Golley RK, Mittinty MN, Smithers LG, Emmett P, Northstone K, et al. Diet spanning infancy and toddlerhood is associated with child blood pressure at age 7.5 y. Am J Clin Nutr. 2013;97:1375–86.
17. Golley RK, Smithers LG, Mittinty MN, Emmett P, Northstone K, Lynch JW. Diet quality of U.K. infants is associated with dietary, adiposity, cardiovascular, and cognitive outcomes measured at 7–8 years of age. J Nutr. 2013;143:1611–7.
18. Barker M, Lawrence WT, Skinner TC, Haslam CO, Robinson SM, Inskip HM, et al. Constraints on food choices of women in the UK with lower educational attainment. Public Health Nutr. 2008;11:1229–37.
19. Cooper C, Eriksson JG, Forsen T, Osmond C, Tuomilehto J, Barker DJ. Maternal height, childhood growth and risk of hip fracture in later life: a longitudinal study. Osteoporos Int. 2001;12:623–9.
20. Godfrey KM, Gluckman PD, Hanson MA. Developmental origins of metabolic disease: life course and intergenerational perspectives. Trends Endocrinol Metab. 2010;21:199–205.
21. Barker DJP. Mothers, Babies and health in later life. Edinburgh: Churchill Livingstone; 1998.
22. Eriksson JG, Forsen T, Tuomilehto J, Osmond C, Barker DJ. Early growth and coronary heart disease in later life: longitudinal study. BMJ. 2001;322:949–53.
23. Gluckman PD, Hanson MA, Bateson P, Beedle AS, Law CM, Bhutta ZA, et al. Towards a new developmental synthesis: adaptive developmental plasticity and human disease. Lancet. 2009;373:1654–7.
24. Gluckman PD, Hanson MA, Cooper C, Thornburg KL. Effect of in utero and early-life conditions on adult health and disease. N Engl J Med. 2008;359:61–73.
25. Pike KC, Hanson MA, Godfrey KM. Developmental mismatch: consequences for later cardiorespiratory health. BJOG. 2008;115:149–57.
26. Inskip H, Baird J, Barker M, Briley AL, D'Angelo S, Grote V, et al. Influences on adherence to diet and physical activity recommendations in women and children: insights from six European studies. Ann Nutr Metab. 2014;64:332–9.
27. Nutbeam D. The evolving concept of health literacy. Soc Sci Med. 2008;67:2072–8.
28. Ratzan SC. Health competent societies: our challenge and future. J Health Commun. 2009;14:99–101.
29. Siegel H. Why should educators care about argumentation? Informal Logic. 1995;17:159–76.

30. Millar R, Osbourne J. Beyond 2000: Science Education for the Future. King's College London, School of Education;1998.
31. Roberts DA. Scientific Literacy/Science Literacy. In Handbook of Research on Science Education. Edited by Abell SK, Lederman NG. Lawrence Erlbaum Associates Inc: New Jersey, USA;2006:729–80.
32. Baird J, Cooper C, Margetts BM, Barker M, Inskip HM. Changing health behaviour of young women from disadvantaged backgrounds: evidence from systematic reviews. Proc Nutr Soc. 2009;68:195–204.
33. Borzekowski DL. Considering children and health literacy: a theoretical approach. Pediatrics. 2009;124 Suppl 3:S282–8.
34. Nutbeam D. Getting evidence into policy and practice to address health inequalities. Health Promot Int. 2004;19:137–40.
35. Marks R. What works, what is needed, and why? Health Educ. 2009;109:4–8.
36. Ofsted. Learning outside the classroom - How far should you go? 2008.
37. Grace M, Woods-Townsend K, Griffiths J, Godfrey K, Hanson M, Galloway I, et al. A science-based approach to developing teenagers' views on their health and the health of their future children. Health Educ. 2012;112:543–59.
38. Department for Education and Skills. Learning Outside the Classroom Manifesto. London: DfES; 2006.
39. O'Donnell L, Morris M, Wilson R. Education Outside the Classroom: an Assessment of Activity and Practice in Schools and Local Authorities. In: DfES Research Report 803. London: DfEs; 2006.
40. Braund M, Reiss M. Towards a more authentic science curriculum: the contribution of out-of-school learning. Int J Sci Educ. 2006;28:1373–88.
41. Bagust L, Davey H, Woods-Townsend K. LifeLab: Me, My Health and My Children's Health. University of Southampton. 2014.
42. Guttersrud O, Dalane JO, Pettersen S. Improving measurement in nutrition literacy research using Rasch modelling: examining construct validity of stage-specific 'critical nutrition literacy' scales. Public Health Nutr. 2014;17:877–83.
43. Crozier SR, Inskip HM, Barker ME, Lawrence WT, Cooper C, Robinson SM. Development of a 20-item food frequency questionnaire to assess a 'prudent' dietary pattern among young women in Southampton. Eur J Clin Nutr. 2010;64:99–104.
44. Linnan L, Steckler A. Process Evaluation for Public Health Interventions and Research. San Francisco: John Wiley and Sons; 2002.
45. Moore G, Audrey S, Barker M, Bond L, Bonell C, Cooper C, et al. Process evaluation in complex public health intervention studies: the need for guidance. J Epidemiol Community Health. 2014;68:101–2.
46. Moore GF, Audrey S, Barker M, Bond L, Bonell C, Hardeman W, et al. Process evaluation of complex interventions: Medical Research Council guidance. BMJ. 2015;350:h1258.
47. Johnson BT, Scott-Sheldon LA, Carey MP. Meta-synthesis of health behavior change meta-analyses. Am J Public Health. 2010;100:2193–8.
48. Bay JL, Mora HA, Sloboda DM, Morton SM, Vickers MH, Gluckman PD. Adolescent understanding of DOHaD concepts: a school-based intervention to support knowledge translation and behaviour change. J Dev Orig Health Dis. 2012;3:469–82.
49. Capewell S, Graham H. Will cardiovascular disease prevention widen health inequalities? PLoS Med. 2010;7:e1000320.

Submit your next manuscript to BioMed Central and take full advantage of:

- **Convenient online submission**
- **Thorough peer review**
- **No space constraints or color figure charges**
- **Immediate publication on acceptance**
- **Inclusion in PubMed, CAS, Scopus and Google Scholar**
- **Research which is freely available for redistribution**

Submit your manuscript at
www.biomedcentral.com/submit

BioMed Central
