# Supplementary material for: A cluster-randomised controlled trial of the LifeLab education intervention to improve health literacy in adolescents
Source: PLoS One. 2021 May 5;16(5):e0250545. doi: 10.1371/journal.pone.0250545 (PMC8099135; doi:10.1371/journal.pone.0250545)
Supplement: Attachment — Submitted filename: Response to Reviewers PONE-D-19-24269.docx [file pone.0250545.s007.docx]

Joerg Herber

Editor-in-Chief

PLOS ONE

5^th^ October 2020

Dear Dr Herber,

**Re: Manuscript reference number: PONE-D-19-24269**

**Title:** **A cluster-randomised controlled trial of the LifeLab education intervention to improve health literacy in adolescents**

We are very grateful to the Editors and the Reviewers for their helpful and supportive comments. We have addressed all their points below and have attached a revised version of our manuscript with tracked change. We have reproduced the editors’ and reviewers’ comments in bold below and added our responses in regular non-italicised font indicating how we have altered the paper to address the comments. Excerpts from the manuscript are also provided in italicised font with corresponding line numbers.

We hope you find our edits favourable and that the manuscript is now acceptable for publication.

Yours Sincerely,

Kathryn Woods-Townsend

**Journal Requirements:**

1) **Please ensure that your manuscript meets PLOS ONE's style requirements, including those for file naming. The PLOS ONE style templates can be found at**

The manuscript has been edited to meet PLOS ONE’s style requirements.

2) **Thank you for including your competing interests statement; "I have read the journal's policy and the authors of this manuscript have the following competing interests:
   KMG has received reimbursement for speaking at conferences sponsored by nutrition companies, and is part of an academic consortium that has received research funding from Abbott Nutrition, Nestec and Danone. The University of Southampton has received an unrestricted donation from Danone Nutricia to support LifeLab’s work with schools."**

**Please confirm that this does not alter your adherence to all PLOS ONE policies on sharing data and materials, by including the following statement: "****This does not alter our adherence to PLOS ONE policies on sharing data and materials.” (as detailed online in our guide for authors** [**http://journals.plos.org/plosone/s/competing-interests**](about:blank)**).  If there are restrictions on sharing of data and/or materials, please state these. Please note that we cannot proceed with consideration of your article until this information has been declared.**

We have included the required wording (lines 538-539)

3) **Please include your updated Competing Interests statement in your cover letter; we will change the online submission form on your behalf.**

We have included the updated Competing Interests statement in our cover letter.

4) **Thank you for submitting your clinical trial to PLOS ONE and for providing the name of the registry and the registration number. The information in the registry entry suggests that your trial was registered after patient recruitment began. PLOS ONE strongly encourages authors to register all trials before recruiting the first participant in a study.**
5) **As per the journal’s editorial policy, please include in the Methods section of your paper:**
i. **your reasons for your delay in registering this study (after enrolment of participants started);**

We have included the following text to explain our delay in registering this study (lines 176-180):

*“The concept for this project was informed by our pilot trial[1] and developed in 2014. In the initial phases, as this was an education RCT, the requirement to register as a clinical trial was not appreciated. Consequently, although the trial registration process was initiated in September 2014, final confirmation of registration did not happen until March 2015.”*

ii. **confirmation that all related trials are registered by stating: “The authors confirm that all ongoing and related trials for this drug/intervention are registered”.**

We have included the required wording (lines 180-181).

iii. **Please also ensure you report the date at which the ethics committee approved the study as well as the complete date range for patient recruitment and follow-up in the Methods section of your manuscript.**

We have included the date of ethics committee approval (lines 172-173) and the complete date range for participant recruitment and follow-up (lines 197-199).

6) **Thank you for including your ethics statement on the online submission form: "The study was approved by the Research Ethics Committee of the Faculty of Social Sciences, University of Southampton (ERG reference: 7892 amendment 1 (10/10/13), ERG reference: 12328 (14/11/14) and amendment 18817 (14/01/2016). Written consent was obtained from participants and their parents.". To help ensure that the wording of your manuscript is suitable for publication, would you please also add this statement at the beginning of the Methods section of your manuscript file.**

We have added this statement to the beginning of the Methods section in the clean manuscript file.

7) **In line with our goal of ensuring long-term data availability to all interested researchers, PLOS’ Data Policy states that authors cannot be the sole named individuals responsible for ensuring data access (https://journals.plos.org/plosone/s/data-availability#loc-acceptable-data-sharing-methods).**

**Before we proceed with your manuscript, please address the following prompts:**

i. If there are ethical or legal restrictions on sharing a de-identified data set, please explain them in detail (e.g., data contain potentially identifying or sensitive patient information, data are owned by a third-party organization, etc.) and who has imposed them (e.g., a Research Ethics Committee or Institutional Review Board, etc.).
ii. Please also provide contact information for a non-author entity to which data requests may be sent, such as your institution's data access committee, ethics committee, or other institutional body.
iii. If there are no restrictions on sharing an anonymized dataset, please upload the minimal anonymized data set necessary to replicate your study findings to a stable, public repository and provide us with the relevant URLs, DOIs, or accession numbers. For a list of recommended repositories, please see https://journals.plos.org/plosone/s/recommended-repositories. You also have the option of uploading the data as Supporting Information files, but we would recommend depositing data directly to a data repository if possible.

Thank you for this request. We have uploaded the de-identified data used in the analysis presented in this manuscript, The DOI is: <https://doi.org/10.5258/SOTON/D1606> and will be activated upon acceptance of the manuscript.

In the process of de-identifying the data and preparing for upload to the repository, we have identified one participant who only took part in the 12month follow-up and have deleted their record, but this means that the number in the study has reduced from 2930 to 2929 – Table 1 and Fig2 have been amended to reflect this. Furthermore we have identified a small error in Table 3, and have amended the numbers accordingly.

**Reviewers' comments:**

**Reviewer #1:**

**This is a model cluster randomized trial. There are a few statistical concerns that should be addressed in a revision:**

1) **The randomization procedure used appears to be a "random block design" according to the textbook (Rosenberger and Lachin, Randomization in Clinical Trials". It appears that blocks were randomly selected from size 2 to 12, yet there are only 38 schools, so it is not clear how many blocks were selected, and why the large number 12 was the upper limit. And were blocks sizes chosen uniformly among 2,4,6,8,10,12? Not clear at all.**

We have added the following text to lines 211-214: “*The blocks were not randomly selected as we needed to accommodate the needs of the schools to be randomised quickly. We obtained the largest even number of schools we could in a short space of time and then randomised. The block sizes varied between 2 and 12.”*

2) **The sample size is relatively small, justified by an intraclass correlation and measures of variability. Yet there is not mention in the discussion of whether the sample size assumptions were realized in the study? Was the sample size actually adequate? If not, what was the actual power for the primary outcome?**

We have added the final intraclass correlation to the results section of the paper (lines 349-350), and checked the assumptions of normality. Post hoc sample size calculations are not generally advised as they are biased (For a recent reference on the topic see: Dziak JJ, Dierker LC, Abar B. The Interpretation of Statistical Power after the Data have been Gathered. Current psychology (New Brunswick, NJ). 2020;39(3):870-7.) Historically, such power calculations have been conducted when the observed effect size is smaller than that used in the original power calculation and the aim is to see what power there would be for that reduced effect size. In our case, the observed effect size was slightly larger than that used in the original power calculation and the p-value for the primary outcome was less than the α in the original power calculation, so this trial was not underpowered. As a result, we have not amended the text and hope that this is acceptable.

3) **More information on the assumptions of the model, including the distributional assumption of the random effect, and appropriate residual analysis and diagnostics are needed, as would be required by any regression class.**

We have added the word ‘*linear*’ to line 294 to describe the model more accurately and given details in lines 298-299 of the Poisson model with a log link that was used for the binary outcomes to estimate prevalence rate ratios. We have stated that the random effects were assumed to be independently normally distributed (lines 306-307) and described the diagnostic methods used for assessing the fit of the model (lines 307-309) and the results of those diagnostic tests in lines 347-349.

4) **In general, statistical methods for cluster randomized trials are very different from others, and appropriate statistical references should be made (e.g., Donner and Klar, Cluster Randomized Trial among many others).**

We have added a reference (line 294) to the book by Hayes and Moulton on Randomised Clinical Trials, which is the book that we used.

**Reviewer #2:**

**General Comments**

**This is an interesting and useful article. By focussing on a non-clinical, adolescent population, it provides much-needed and useful evidence and authors should be commended on the reach and retention rate. Given the primary outcome is theoretical healthy literacy, readers need to be mindful that this is measured by the use of a non-validated (albeit soundly developed) questionnaire. This is clearly indicated by the authors and this paper should not be stalled by this. There was and is no such measurement tool yet available, and the questionnaire used was therefore appropriate. The large sample size is to be commended, both in terms of adolescent participants, and number of schools involved. The paper presents practical and feasible methods to improve health literacy in adolescents and is a welcome contribution to our knowledge in this area.**

We thank the reviewer for their supportive and insightful comments, reflecting much of how we felt during the design and delivery of this trial.

**Overall comments**

1) **The primary outcome was theoretical health literacy. Although line 395-402 details that no validated health literacy questionnaire for this age group currently exists, I think this should be more clearly signposted in the introduction to justify why the authors developed their own questionnaire. The use of feasibility and pilot studies to inform the questionnaire development should also be stressed earlier in the paper, to support the rigorous approach adopted by the authors.**

We agree with the reviewer that there is a lack of a validated tool to measure health literacy in this age group and take on board the need to signpost this in the Introduction. We have added the following text to lines 112-118 “*Currently there are no validated instruments for assessing health literacy in this population. Consequently, during the development work for this trial, building on work carried out by Guttersrud et al [22], but considering issues that were more appropriate for adolescents, a series of health literacy questions were identified. These have been used previously in feasibility and pilot studies [18, 23] and have been shown to be acceptable and understood by this age group, giving measurable differences over time.*”

2) **Methods/Results. Well written, and clear methodology and stat analysis sections. Statistics and results seem to be well presented also - though I say this with the caveat that my statistics knowledge is passable, but not expert. I would advocate however for including cross sectional baseline data (results) from the questionnaire - data that isn’t that readily available internationally. This should then be discussed and contextualised in the discussion section. If this data has been published elsewhere in it’s own right, then it can be referred to again here - but important for the reader to at least get a grasp of the findings cross sectionally for the population, and what this means in the context of international literature.**

We agree with the reviewer that cross sectional baseline data are important – these are included in tables 2 and 3 and we draw attention to some key findings in the results section, with the addition of the following text – lines 361-366: “*At baseline, adolescents in both groups showed a high level of agreement with the question “The food I eat now may affect my health in the future” but were less likely to understand the impact of parental diet (particularly father’s diet) on the health of future children. The questions relating to impact of parental diet on future generations showed greater change for intervention participants.”* There was also discrepancy in awareness of the ability to lower risk for heart disease and cancer, as noted in lines 380-382.

3) **Very short discussion offered, I would suggest that the Discussion be extended to more fully consider other relevant or similar programmes**

We have added in a description of other relevant programmes to line 428-436 “*Comparison with other studies:*

*The development of LifeLab drew on the experience of a similar laboratory-based facility for schools in Auckland, New Zealand, known as LENScience. Many children from across the country visited LENScience, and a before- and after- comparison was undertaken, albeit without a control group. Similar to our findings, the students’ knowledge increased, but they also showed some limited impact on behaviour change [[21]. However, conducting such studies at a time when adolescents are undergoing rapid changes in their behaviours means that the lack of a contemporaneous control group makes interpretation of such findings difficult. This was the rationale for the present study.”*

4) **and also to take into account the fact that there didn’t exist a validated questionnaire for HL of adolescents.**

We have discussed the lack of appropriate tools to measure health literacy in this population in the discussion and following reviewer’s comments have added additional text into the introduction to signpost to this earlier in the manuscript – we hope that this is sufficient to address this comment.

5) **IN addition, consideration of what was found cross sectionally for the entire cohort warrants presentation and then discussion here - probably at the opening of this Discussion section. What did you find out from these students? and what does it tell us about HL? The change that you detected, how well does it stand up to other studies? What was the magnitude of this change in terms of context? Etc**

Thank you for the comment. We have added the following text in the results section (lines 361-366) “*At baseline, adolescents in both groups showed a high level of agreement with the question “The food I eat now may affect my health in the future” but were less likely to understand the impact of parental diet (particularly father’s diet) on the health of future children.”* to reflect the finding, that whilst the participants showed good awareness of the impact of their own diet on future health, they were less aware of the impact of parental diet on future children’s health. In terms of what this tells us about health literacy in this population, few studies have looked at health literacy in adolescents, so there is little to compare our data to. We hope that this study offers data to which others can compare their data to.

The change detected showed an increase in knowledge but this didn’t correspond in these measures to change behaviour – as noted. This is the challenge for public health interventions of this type, leading to our recommendations to consider further intervention elements.

6) **Please see attached word document for tabulated line by line recommendations/changes required.**

| **Comment number** | **Pg** | **Line** | **Comment** |
| --- | --- | --- | --- |
| **1** | **3** |  | **I would encourage authors to consider removal of the first person from the language of the paper in favour of the third person, but particularly so in the abstract. In general I would encourage more ‘scientific’ language, but again this is most particularly relevant in the abstract, for example:**   - **‘We evaluated whether an educational intervention, ...’ - change to ‘This study was carried out to evaluate whether an educational intervention which …’** - **‘The primary outcome was change in theoretical health literacy from before to 12 months after intervention’ change to ‘The primary outcome was change in theoretical health literacy from pre- to 12 months post-intervention’.** |
| We have incorporated those recommended changes to the text in the abstract (lines 53-54, lines 56-57 and line 66 | | | |
| **2** | **4** | **103** | **Sentence does not read well consider revising** |
| We have re-written this sentence to provide clarity, lines 104-110 *”Lifelab is based on the premise that health and wellbeing are socio-scientific issues [17], that is, social issues with prominent scientific components [18, 19]. The LifeLab curriculum aims to foster an understanding of socio-scientific knowledge, alongside decision-making skills, thereby promoting the adolescents’ sense of control over their lives and futures. It is suggested that one means of increasing health literacy is by developing scientific literacy; this is achieved by providing education for adolescents in ‘science for health literacy’ [20].”* | | | |
| **3** | **5** | **118** | **Can authors provide any additional evidence of existing interventions in relation to health outcomes beyond obesity and physical activity?** |
| Additional references from systematic reviews showing the inconsistent impact of the effectiveness of school-based interventions on health outcomes (healthy eating, mental health, oral health) have been included – lines 126-127 | | | |
| **4** | **5** | **123-126** | **Sentence does not read well consider revising** |
| We have re-written this sentence to provide clarity, lines 137-141 *“The LifeLab curriculum has been designed to be embedded within the school curriculum. It is an innovative, ‘hands-on’, educational intervention, which aims to promote health literacy through science engagement and increasing scientific literacy (see the LifeLab panel and TIDIER framework check list (S1) for further detail)[23].”* | | | |
| **5** | **6** | **152** | **Panel is a really clear way of explaining the intervention components** |
| Thank you, we are glad that this is a useful way of presenting the intervention components. | | | |
| **6** | **7** | **156** | **Says 32 secondary schools were recruited but Abstract says 38 (pg2, line 56) . The reason for this later becomes clear at pg 8 (line 182), but I think it should read 38 at line 156 for consistency.** |
| Number of schools recruited has been edited for clarity (line 168) | | | |
| **7** | **8** | **167** | **Indicate number of single sex schools as this is adjusted for in analysis (line 255, pg 10).** |
| Thank you for this comment; of the 38 schools recruited to the trial, only 1 school was single sex and so we felt it necessary to adjust for gender at individual level, but not to consider at school level. Due to the representation of one single sex school in our participants, we feel it could potentially jeopardise anonymity to state this and so have not included this detail. We hope this explanation is acceptable. | | | |
| **8** | **9** | **208** | **Who decided which issues were more appropriate for adolescents? Could examples be provided?** |
| Thank you for this comment, we have re-written this sentence to provide more clarity – lines 243-244: “*guided by our previous pilot studies and PPI input from young people themselves*” | | | |
| **9** | **10** | **253** | **Remove ‘our’** |
| Removed. Line 300 | | | |
| **10** | **11** | **272** | **Final column heading of Table 1 should read national average** |
| Thank you for highlighting this, this has been corrected. | | | |
| **11** | **9** | **199 - 205** | **In this section include detail on how long teh questionnaire took to complete, where in the school generally they were administered, how large the class sizes were on average, the ratio of researcher to student, and how literacy issues were managed.** |
| Thank you for the request for additional clarity around administration of the questionnaires. We have added the following text for clarity, line 225: “*typically a science lesson*” and lines 231-236: “*The questionnaire took on average 20 minutes to complete. Students either completed the questionnaires on iPads brought into school as part of the data collection exercise, on school laptops or devices, or in a school computer room. Typically, classes consisted of 30 students, with the class teacher and a researcher present. Students with literacy issues were supported by the class teacher or researcher reading out the questions to them.”* | | | |
| **12** | **11** | **263-265** | **Remove these lines to include in methods rather than results** |
| This detail is now included in the methods section, lines 197-199 | | | |
| **13** | **12** | **284** | **‘There were small differences’ - need to contextualise this do you mean ‘demographic differences?** |
| Thank you for this comment. In answer to Reviewer 3’s third comment, we have provided more detail and rewritten this sentence (lines 335-341): *“In the control group 12.8% (95%CI: 11.2 to 14.6%) of those completing the baseline questionnaire did not complete the follow-up while the corresponding figures for the intervention were somewhat higher 17.7% (95%CI: 15.7 to 19.8%). Intervention schools had a more disadvantaged profile than control schools; as a consequence the number of intervention participants living in more deprived areas who dropped out of the study was also higher.”* | | | |
| **14** | **15** | **330** | **Full stop after intervention. Change ‘they’ to ‘adolescents’** |
| Edited to include these changes – lines 394-395 | | | |
| **15** |  | **332-335** | **Add citations to support this sentence** |
| We have added the following sentence and citation to the COM-B model of behaviour change – lines 396 – 401 **“***The COM-B (capability, opportunity and motivation) model of behaviour change proposes that capability encompasses knowledge, which is one precursor for changes in behaviour [44].* *The experiential learning opportunities provided by the LifeLab programme, increased adolescents’ knowledge and enabled them to access, understand and reflect on what they need to do to live healthier lives; this was demonstrated in their improved health literacy at follow-up***.***”* | | | |
| **16** |  | **341 - 343** | **Sentence needs a little more explanation, and possibly link to literature that has cited such ‘upstream determinants’ previously** |
| Thank you for your comment. We have added a further sentence to provide clarity, along with additional citations. We hope this explains our reasoning more clearly. Lines 408 – 417: *“It has been suggested that for public health interventions to be effective they need to focus on upstream determinants of obesity, such as the environments in which people live; the physical, socio-cultural, economic and political contexts that influence diet and physical activity levels [25, 47, 48]. These upstream determinants are largely beyond the control of the individual; thus an environment that is conducive to healthy choices is essential to support optimal individual choice [48]. Our work suggests that education, specifically an individual’s scientific and health literacy education, could be seen as a potential, but less conventional, upstream determinant which should be considered in the design and delivery of public health interventions.”* | | | |
| **17** | **15** | **333** | **Comma after behaviour. Suggest reference is needed to support such a crucial point** |
| This sentence has been revised following point 15 above. | | | |
| **18** | **15** | **346-353** | **Long sentence consider revising** |
| Thank you for your comment. This sentence has been rewritten to provide clarity- Lines 421 – 427: *“Recent evidence shows that the rate of obesity-related cancers is increasing more rapidly in young adults [49]. There is also a lack of awareness of the link between factors such as poor diet, sedentary behaviours and excess weight and risk of cancer [50]. As such, there is clearly a need to ensure that the correct messages are embedded in the education system [51], and importantly that there is a focus on improving the health literacy of adolescents.”* | | | |
| **19** | **16** | **370** | **Could further evidence be presented to support unpublished systematic review** |
| This systematic review has been reviewed and reviewers’ comments addressed, so we are hopeful this will be published shortly. However, to address this comment, additional text has been included – lines 454-458: *“Behaviour change frameworks highlight the importance of a trusted ‘facilitator’ to deliver the intervention [54] and earlier research shows that school students prefer interventions delivered by teachers [8, 55]. Systematic reviews have shown that training for teachers prior to delivery of interventions is associated with effectiveness of the intervention [56] and Jacobs et al, under review].”* | | | |
| **20** | **17** | **408** | **‘Professionals’ or teachers or those experienced in working with this age group? This may not necessarily be class teachers, but any qualified teacher, this is an important point and should be made clearer.** |
| Thank you for this comment. We agree that this is an important point and so have added some additional text to emphasise the point – lines 510-514: *“education professionals, either subject or class teacher, or other suitably qualified educator, who has an understanding of the pedagogical approaches best employed to engage with the target age groups. This will also ensure that the intervention can be situated within the curriculum that the young people are following – reinforcing prior knowledge and showing the relevance to other aspects of their learning.”* | | | |
| **21** | **17** | **415** | **Unsure how the recommendation for ‘digital technology’ fits in relation to the study findings. May fit better within the strengths and limitations section.** |
| Thank you for your comment. We are trying to convey, that whilst we have shown change in health literacy, to translate this to change in behaviour may/will require additional intervention elements, and digital technology, particularly for this age group may be the answer. So, we have slightly re-written this sentence to provide clarity, but retained this point in this section of the manuscript – we hope this is acceptable. Line 519 – 523: *“Future work to capitalise on this and support changes in adolescent health behaviour may require the addition of other intervention elements. For example, digital technology may provide an opportunity for delivering tailored, responsive and engaging interventions in a form that may be particularly appealing to adolescents”*  *We have also added the following text to the Strengths and Limitations section to reflect the situation, that this intervention may require additional elements to translate impact into behaviour change – lines 501-504: “This intervention showed change in knowledge, which, in the outcome measures selected did not translate into behaviour change; this limitation of the current trial could reflect a need to include additional intervention elements which support participants to effect change in behaviour.”* | | | |

**Reviewer #3:**

**It’s very rare to see published RCTs of school-based health interventions, specifically cluster randomised trials, and as such, this article makes a valuable contribution to the literature. The choice of methodological approach, though challenging to implement, is appropriate, and the authors give a good justification of why blinding is not possible. Aside from the trial, documenting the innovative work of the ‘LifeLab’ programme is another good reason for publishing this article, and the groups desire to gather quality evidence of impact, emphasises their commitment to quality implementation.**

Thank you for your comments and recognition of the importance of conducting this type of research in schools.

**Having said this there are a few areas where the paper could be strengthened as discussed below:**

1. **Given previous LifeLab studies which shared evidence of the benefits of participation to students, an ethical question raised by the study is: what benefit did control schools gain from participating in the study? For example, were control schools included in the implementation in subsequent years? Or did they benefit in any other way, in order to balance the time taken for surveys? I appreciate that offering a different intervention for the controls, though potentially beneficial to the students, may compromise a clean comparison, however a sentence to justify this or how control schools benefitted from participation would be helpful.**

Thank you for this comment, and apologies for the oversight. This indeed is a concern of ours, and consequently, control schools are given priority for participating in the LifeLab programme in subsequent academic years. This has proved an acceptable solution to schools, and all schools are happy to participate and be randomised, fully understanding the consequences of being randomised to the control arm. We have included an additional sentence to clarify this – lines 238-240: *“, as set out in Panel 1.* *Control schools received normal schooling, they were offered the chance to attend LifeLab in the subsequent year, with a different cohort of students.”*

**2.	Don’t the overlapping confidence intervals of baseline characteristics (disadvantage indicators) between control and intervention schools, suggest that the differences between arms are not statistically significant? Please clarify.**

We have not tested the differences in baseline characteristics between control and intervention as any differences are random. It is an established principle in randomised controlled trials that testing for baseline differences is inappropriate as any differences are random a priori. We have however, clarified the headings of table 1 and hope that this helps. As part of this edit, we realised that when presenting the school characteristic data, we had omitted to include the data from the school which dropped out following recruitment. As we were following an intention to treat protocol, we have updated the intervention school characteristics to include this information – consequently the school characteristic data has changed slightly, but does not change our interpretation of results. This does not affect student characteristic data, as no students from this school participated in data collection

3. **Overall participation/withdrawal/absence/declining data are inconsistently presented within the text of the findings section (lines 280-283). While ‘not consenting’ is given for both arms at baseline, the comparison between control and intervention for absence, withdrawal etc. has not been presented. Including these with their confidence intervals for the arms would allow the reader to judge statistical significance.**

Thank you for your comment. We have added the proportions that did not complete the follow-up and the confidence intervals in lines 335-338 as follows “*In the control group 12.8% (95%CI: 11.2 to 14.6%) of those completing the baseline questionnaire did not complete the follow-up one while the corresponding figures for the intervention were somewhat higher 17.7% (95%CI: 15.7 to 19.8%).”*

4. **The repeated absence of 9.8% looks quite high. How does this compare to national figures? And is there a difference between intervention and control? (this is just a suggested point of discussion as opposed to a recommendation)**

Thank you for this comment. Looking at government data for pupil absence in schools in England, this isn’t a high figure (<https://www.gov.uk/government/statistics/pupil-absence-in-schools-in-england-autumn-term-2017>). Looking specifically at the years over which this RCT was conducted: in 2014, the national figure for secondary schools was 14%, in 2015, it was 12.1% and in 2016, it was 13.4%. There was a difference between control and intervention participants, with more intervention participants being persistently absent (11% vs. 7.5%) – this aligns with our analysis showing that the intervention participants were from slightly more deprived areas. We have clarified in the text the differences between control and intervention schools and specifically in relation to follow-up rate – lines 335-341. We have also added the following text to the strengths and limitations section – line 470-473: *“Although we took a cluster randomisation approach, the relatively small numbers to include as individual clusters (n=38) led to some imbalances, notably the follow-up was poorer in intervention schools which were in more deprived areas and this has to be considered a possible bias in interpreting the results”*

5. **It’s noteworthy that responses to ‘The food I eat now may affect my health in the future’ did not change for both arms while statistically significant PRRs were observed for the other questions in table 2. At baseline, there was high agreement to this statement (The food I eat) for both arms, which suggests a good general background awareness of the impact of diet on individual health… but less so on the impact of parental diet on future generation health. The intervention appears to have had a modest and statistically significant impact on specific awareness in this area and is thus an important finding which could be highlighted in the discussion.**

Thank you for this comment. We agree this is pertinent point that should be drawn out more and so have added additional text to highlight this finding – lines 361-366: “*At baseline, adolescents in both groups showed a high level of agreement with the question “The food I eat now may affect my health in the future” but were less likely to understand the impact of parental diet (particularly father’s diet) on the health of future children. The questions relating to impact of parental diet on future generations showed greater change for intervention participants.”*

6. **With regard to: ‘Specifically, intervention participants were less likely to report their lifestyle as ‘very healthy’ or ‘healthy’ (53.4%) compared with controls (59.5%) at the end of the study (PRR=0.94; 95%CI= 0.87, 1.01)’, that CI’s cross over 1.0 would suggest that though approaching significance, the finding is not statistically significant. This finding is also referred to in the abstract and drawn upon in the conclusion. While this finding is close to statistical significance, it may be more pertinent to reflect on: while the study has demonstrated impact on theoretical literacy, changing student health behaviours require corresponding contextual/societal shifts towards, for example, parental ability and desire and support change in diets and physical exercise, facilities where students can exercise, supportive social norms to changing behaviours etc. These are notoriously challenging to effect change, and are challenging to address through targeting schools alone. This does not in any way detract from the important value and impact of the intervention on theoretical literacy – perhaps the discussion could reflect on this?**

Given the current debate about statistical significance (Amrhein V, Greenland S, McShane B. Scientists rise up against statistical significance. Nature. 2019;567(7748):305-7.), we have commented where there are effect sizes of interest, but haven’t used arbitrary significance levels. We refer the reviewer to this section of the discussion (lines 388-393). If there are particular areas that the reviewer or editor would like us to highlight further, we would be happy to do so.

**7.	The discussion reflects on the trial demonstrating a proof of concept that RCTs can successfully be done in schools – and for this, the team’s rigour in planning and implementing is to be commended. Also commendable is the participation rate of 85%, and it may be worthy of reflection: the team’s careful pre-trial engagement with the schools, and their flexibility in modifying consent processes to accommodate stakeholder views, is likely to have contributed to its success and high participation/retention rates.**

Thank you for your recognition of the difficulties of conducting RCTs in schools. We have added the following text to further highlight factors which contributed to the successful recruitment and implementation of the RCT – lines 441-442: “*This is also a consequence of the project being embedded within the local education community and good working relationships being cultivated over many years.”*

**Reviewer #4:**

**This is an important study given the lack of research on health literacy in children. The study strengths include the large sample, novel intervention, and potential impact on school curriculum focus on health literacy. However, key methodological details have been omitted.**

**Abstract
Page 3: Line 53, “enable preparation for parenthood” might be too large of a stretch based on the results. Can be re-worded to say “and possibly passing on good health prospects to future children.” This point also relates to the 1st paragraph on page 4. Developmentally, adolescents are more concerned with immediate gratification and preparing for parenthood is far-fetched for them.**

Thank you for this comment. We feel quite strongly that evidence points to adolescence as being a critical time point during which to engage young people about preconception health – see e.g.

- Patton et al. Our future: a Lancet commission on adolescent health and wellbeing 2016;387(10036):2423-78,
- Patton et al. Adolescence and the next generation. Nature. 2018;554(7693):458-66.
- Barker M, et al. Intervention strategies to improve nutrition and health behaviours before conception. Lancet. 2018;391(10132):1853-64.

So, we would prefer to retain these words, but have added the following text to lines 52-53: *“and enable preparation for parenthood – albeit a long way off”*

In relation to paragraph 1 on pg 4, whilst we accept that developmentally adolescents are typically concerned with their imminent futures, they are engaged with concerns with long-term consequences – as per the current movement around climate change. They are also motivated by thinking of the futures of others – as per the success of second-hand smoke campaigns in reducing smoking. This is a key aspect to our work – engaging the students with considering the impact of life choices on future children.

**Introduction**

**Page 4: Are there any studies showing how scientific literacy or science for health literacy is linked to adolescents’ behaviors? If so, please include**

Thank you for this comment; it is a valid point, and whilst there are many references to change in attitudes, the next step of seeing change in behaviour has less research basis currently. However, we have included the following text and citation as support – Lines 111-112: *“Research is emerging, that considers the effectiveness of such interventions on adolescents’ behaviour [21].”*

**Page 5: Expand on “inconsistent findings” in line 117 and “limited success” in line 119.**

We have re-written this sentence for clarity – lines 125 – 132: *“Effects on health outcomes such as levels of obesity [26,26], healthy eating [27], oral health [28], mental health [29] and physical activity [30] have been inconsistent, some showing positive effects, but some having no impact. Interventions targeted at adolescents (aged 10-19) [31] have also had limited success in achieving their outcomes, possibly because these tend to adopt an instructional, transmission approach, simply providing information to students [32,33].”*

**Page 5: Lines 116 to 121, it is unclear whether the authors are referring to health behavior** **interventions in general or scientific literacy interventions specifically.**

We have added the following text for clarity – line 124: *“School-based interventions, aimed at impacting health outcomes have, however, mostly been aimed at younger children rather than adolescents.”.*

**Page 7: Figure 1, under component 2, a sample outline of one of the lessons in the module would be help readers visualize the intervention.**

Thank you for the comment. We have added the following text to Panel 1 to give context to the module of work: *“The over-arching theme for the module of work looks at ‘How scientists work’ – how research questions are formulated, how studies are designed, how data are collected, analysed and communicated. The lessons are based on real-life research studies, such as the Southampton Women’s Survey [38].”*

**Method**

**Page 9: Given that health literacy is the primary outcome. More information on the measure is warranted (e.g., how many items, response options, measurement statistics, how was the measure developed and validated in the pilot RCT, how does the measure map on to the intervention objectives) in this section. The reader should not need to scroll to the results table to figure out number of items and response options.
- Please report on the overall mean and std deviation of the total health literacy score and the individual items.**

Thank you for this comment; we agree that more information would be helpful and so have given more detail in the methods section to provide clarity – lines 247-257: *“**These were a series of five questions, four of which used a Likert scale, with five responses ranging from ‘strongly disagree’, (scored 1), to ‘strongly agree’ (scored 5), the fifth question “At what age do you think our nutrition starts to affect our future health?” has responses from before birth then in decades up to > 60 years.  These were coded as >30 years = 1, 20-30 = 2, 10-20 = 3, 0-10 = 4, Before birth = 5. Responses to the individual health literacy questions were coded and totalled, and the scores ranged from 6 to 30, with 30 being the highest score possible. The baseline scores were then standardised to produce a normally distributed theoretical health literacy score with a mean of 0 and a standard deviation of 1, with higher scores indicating better health literacy.  The follow-up scores were standardised to the mean and standard deviation of the baseline scores to allow change to be assessed.”*

The health literacy measure was developed and used in our pilot RCT. The measure used in the pilot included more questions; we identified those that were more acceptable and meaningful and discriminated responses. We have acknowledged (lines 498-500) that more work is necessary, as there is no gold standard against which to compare this measure.

**Page 9: Line 201, what kind of support was provided to teachers? Please provide examples and specify if this support was equal across sites.**

Thank you for this comment> We have added the following text for clarity – lines 227-231: *“The LifeLab team supported teachers from all schools during the process of administering the questionnaires; a member of the LifeLab team was present but the students completed the questionnaires independently on their computers. During follow-up data collection, support was again provided as requested by the class teacher.”*

**Results**

**Page 11: Please include other relevant student characteristics (e.g., race and/or ethnicity) to corroborate and illustrate what the authors mean by “broadly representative of the general population” in line 185**.

Thank you for this comment. Whilst the participating schools were broadly similar in terms of the ‘English as an additional language’ (EAL) measure, we did not collect ethnicity data. As can be seen from Table 1, whilst the schools were broadly representative of the schools in the recruitment area, the area as a whole is less ethnically diverse than would be expected for the UK.

**Page 12: Completion statistics for intervention is missing – how many did not complete, average dose of intervention, outliers with school with very low/high dosage and how were these characteristics adjusted for in the analyses? Intervention fidelity – what were the results of the process evaluation?**

Thank you for the comment. As detailed in Figure 2, 247 students did not complete the intervention. All schools had access to the same dosage (Teacher professional development day, including behaviour change training, in-school teaching resources and lessons and the LifeLab activity day). 127 students did not participate in the LifeLab activity day, and so had a lower dose of the intervention. In terms of intervention fidelity – that is the subject for the process evaluation to be addressed in a subsequent manuscript.

**Page 12: Lines 284-285, were these ‘small differences’ statistically significant? Please provide percentages to be consistent with how information is presented in the rest of the paragraph.**

Thank you for this comment. In line with previous reviewers’ comments, this sentence has been re-written to include this data –lines 335-341: *“In the control group 12.8% (95%CI: 11.2 to 14.6%) of those completing the baseline questionnaire did not complete the follow-up one while the corresponding figures for the intervention were somewhat higher 17.7% (95%CI: 15.7 to 19.8%). Intervention schools had a more disadvantaged profile than control schools; as a consequence the number of intervention participants living in more deprived areas who dropped out of the study was also higher.”*

**Discussion**

**Page 15: Lines 340-341, define upstream determinants and expound on how health literacy can be conceptualized and treated as an upstream determinant in the context of this study.**

Thank you for this comment. In line with previous reviewers’ comments, this sentence has been re-written to include the following text for clarity – Lines 408 – 417: *“It has been suggested that for public health interventions to be effective they need to focus on upstream determinants of obesity, such as the environments in which people live; the physical, socio-cultural, economic and political contexts that influence diet and physical activity levels [25, 47, 48]. These upstream determinants are largely beyond the control of the individual; thus an environment that is conducive to healthy choices is essential to support optimal individual choice [48]. Our work suggests that education, specifically an individual’s scientific and health literacy education, could be seen as a potential, but less conventional, upstream determinant which should be considered in the design and delivery of public health interventions.”*
